# Unusual ectopic ACTH syndrome in a patient with orbital neuroendocrine tumor, resulted false-positive outcome of BIPSS:a case report

**DOI:** 10.1186/s12902-020-00590-9

**Published:** 2020-07-31

**Authors:** Huiwen Tan, Dawei Chen, Yerong Yu, Kai Yu, Weiming He, Bowen Cai, Su Jiang, Ying Tang, Nanwei Tong, Zhenmei An

**Affiliations:** 1 Division of Endocrinology and Metabolism, West China Hospital, Sichuan University, Chengdu, 610041 People’s Republic of China; 2 Division of Ophthalmology, West China Hospital, Sichuan University, Chengdu, 610041 People’s Republic of China; 3 Division of Neurosurgery, West China Hospital, Sichuan University, Chengdu, 610041 People’s Republic of China; 4 Department of Pathology, West China Hospital, Sichuan University, Chengdu, 610041 People’s Republic of China

**Keywords:** Cushing’s syndrome, Ectopic Cushing’s syndrome, Orbital neuroendocrine tumor, Bilateral inferior petrosal sinus sampling (BIPSS)

## Abstract

**Background:**

Cushing’s syndrome has been described as a complex endocrine disorder characterized with high cortisol concentration. Correct and early diagnosis of Cushing’s syndrome is challenging. According to the latest guideline, bilateral inferior petrosal sinus sampling (BIPSS) is considered to be the gold standard for the differential diagnosis. However, in some unusual cases, this method may be false positive. Here, we presented a rare case of orbital neuroendocrine tumor secreting adrenocorticotrophic hormone with false positive inferior petrosal sinus sampling.

**Case presentation:**

A 48-year-old woman was admitted to West China Hospital of Sichuan University, presenting with fatigue, whole body edema for 1 year, alopecia and skin pigmentation for 5 months. Hormonal profiles including plasma cortisol and adrenocorticotrophic hormone (ACTH) measurements and low-dose dexamethasone inhibition test suggested that the patient had Cushing’s syndrome. However, during tumor location phase, the results of high-dose dexamethasone inhibition test (HDDST) contradicted desmopressin (DDAVP) stimulation test. Thus, BIPSS was employed, and its results indicated a pituitary origin. Interestingly, MRI of sellar region showed an innocent pituitary but caught a serendipitous lesion in the lateral rectus muscle of left eye, which was later proved to be an orbital neuroendocrine tumor secreting ACTH by pathological and immunohistochemical results. ACTH level of the patients was < 0.1 ng/L and cortisol level was 51.61 nmol/L 1 week after surgery. At 24 months follow-up, the patient appeared stable with no complaints nor any symptoms of Cushing’s syndrome, including moon face, purple striate and central obesity. The patient’s life quality also improved significantly.

**Conclusion:**

We reported a rare case of endogenous Cushing’s syndrome due to ectopic ACTH secreting from an orbital neuroendocrine tumor. This unique case of orbital EAS suggests that orbital venous blood backflow, owning to abnormal anatomic structures, may possibly lead to false positive BIPSS results.

## Background

Cushing’s syndrome (CS) is a complex endocrine disorder characterized by hypercorticosteremia [1, 2]. In terms of etiology, Cushing’s syndrome can be divided into Adrenocorticotropic Hormone (ACTH)-dependent CS and ACTH-independent CS. The former includes pituitary ACTH secreting adenoma [Cushing’s disease (CD)] and ectopic ACTH syndrome (EAS), accounting for 70% ~ 80% of the causes [2, 3]. EAS accounts for approximately 10 to 20% of all cases of CS [2, 3]. The etiological diagnosis of Cushing’s syndrome is important but challenging [4]. According to the latest clinical evidence, MRI cannot be completely relied upon to differentiate between an ectopic and a pituitary origin of ACTH-producing. While simultaneous bilateral inferior petrosal sinus sampling (BIPSS) is recommended to be the gold standard for the differential diagnosis of CD and EAS [5, 6]. However, in some unusual cases, this method may be false positive [7]. Here, we report a case of a rare orbital ACTH-secreting neuroendocrine tumor that lead to false positive BIPSS results.

## Case presentation

A 48-year-old woman, who complained of fatigue, whole body edema for 1 year, alopecia and skin pigmentation for 5 months, was transferred to West China Hospital of Sichuan University on May 25th, 2016. It was noteworthy that the female patient was once hospitalized in her hometown for the orbital mass 20 years ago, but the patient was asymptomatic at that time. Thus the local physician found it to be an inflammatory pseudotumor, and discharged the patient directly without operation. Approximately 1 year before admission, though suffering progressive fatigue, body edema, and palpitation, the patient didn’t apply for any evaluation or treatment till obvious weight gain (10 Kg) was noticed. Then, the patient was admitted to the hospital in her hometown for alopecia, skin pigmentation, and severe fatigue. The blood pressure was 180/116 mmHg, the blood glucose was elevated, and no further treatment approaches were given.

On the patient’s arrival at West China Hospital, additional history was obtained. She didn’t smoke cigarettes, drink alcohol, or use illicit drugs. She didn’t have any surgery history as well. Her menstrual history was basically normal, the age of menarche was 12 years old, and her last menstrual period (LMP) was August 16, 2015. Physical examination showed that the patient had mildly elevated blood pressure (150/79 mmHg), moon face, central obesity, multiple purple striae on abdomen, scattered bruises, and proximal muscle weakness. And her BMI was 25.5 kg/m^2^. And the rest of the physical exam was normal.

**Fig. 1:**
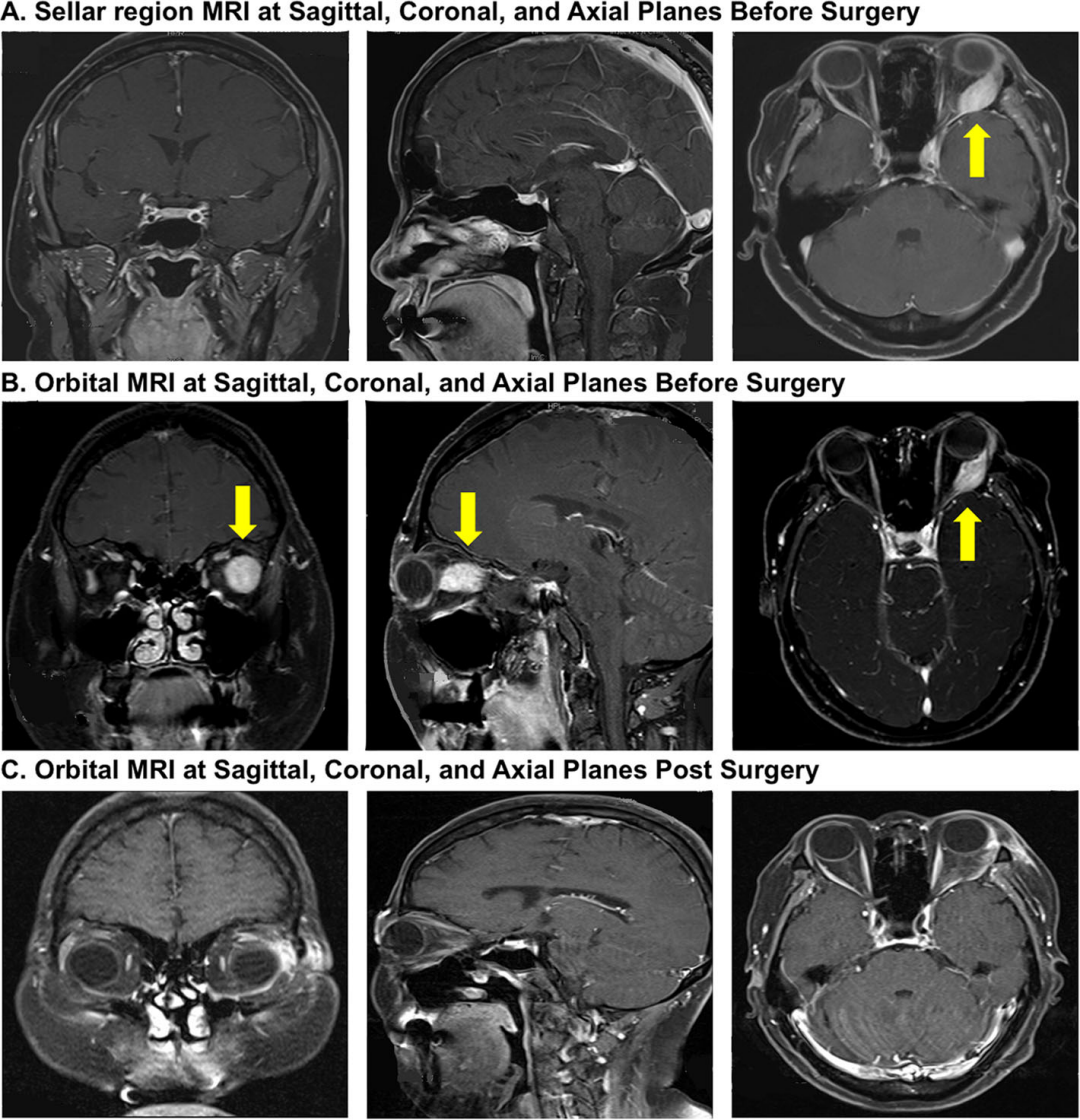
The image profiles of the EAS patient. **a** Pre - operational sellar region MRI shows normal pituitary but orbital mass around lateral rectus muscle of the patient’s left eye. **b** Pre - operational orbital MRI displays a tumorous lesion around lateral rectus muscle of the patient’s left eye. **c** Post - operational orbital MRI shows normal lateral rectus muscle of eye

Laboratory testing showed impaired glucose tolerance (IGT) with fasting blood glucose: 5.7 mmol/L and 2 h blood glucose8.0 mmol/L, negative ketonuria. Islet-specific auto antibodies including GAD -Ab, IA2-Ab, and islet cell antibodies (ICA) were negative. Meanwhile, biochemical results exhibited hypokalemia, but no obvious dysfunctions in patient’s kidney or liver (Table 1). Thyroid function was normal. Initial hormonal measurements showed abnormally elevated levels of cortisol (Cortisol 8 a.m. =887.5 nmol/L, normal range 147.3 ~ 609.3 nmol/L, Cortisol 12 p.m. = 978.6 nmol/L) and morning ACTH concentration (138.1 ng/L, normal range 5.0 ~ 78 ng/L),and the result of 1 mg overnight dexamethasone suppression test indicated Cushing syndrome (Table 2). Then, ACTH measurement, high-dose dexamethasone suppression test were conducted to determine the hidden causes. It showed significant elevation in ACTH level, but failure of PTC suppression, suggesting possible ectopic Cushing syndrome (Table 2). What’s more, pituitary enhanced magnetic resonance imaging (MRI) was negative. However, the profiles of BIPSS summarized in Table 2 contradicted the former results. The blood sampling test of the inferior petroglyphic sinus vein showed that the basic ratio of ACTH in the right inferior petroglyphic sinus to peripheral blood was over 2.0, and the maximum ratio of ACTH in the right inferior petroglyphic sinus to peripheral blood was over 3.0 after the injection of 10μg DDAVP. Moreover, prolactin-normalized ACTH IPS/P ratios and post-DDAVP prolactin-normalized ACTH IPS/P ratios were above 1.3, indicating a pituitary origin. Paradoxical functional tests challenged the diagnosis. Image studies were performed to assist the diagnosis. Chest multi-slice computed tomography (MSCT) was normal. Abdominal CT showed nothing but hyperplastic adrenal glands. Sellar region enhanced magnetic resonance image (MRI) though suggested a normal pituitary, accidentally found an enhancement in the lateral rectus muscle of the patient’s left eye (Fig. 1A). Therefore, orbital enhanced MRI were performed and enhanced orbital mass was found encompassing the lateral rectus muscle of left eye (Fig. 1B). Since the venous communications between ophthalmic vein and inferior petrosal sinus has been reported in several studies [8, 9], we inferred that BIPSS could be false-positive and the orbital mass could be an ectopic ACTH secreting adenoma.

**Table 1:** The abnormal results of biochemical profiles

Items	Results	Normal Ranges	Units
ALT	55	< 40	IU/L
Uric	484	160–380	Umol/L
LDH	397	110–220	IU/L
serum potassium	2.82	3.5–5.5	mmol/L
serum phosphorus	1.64	0.81–1.45	mmol/L

**Table 2:** **The hormonal results and functional measurements of the patient with EAS**

A. Hormone tests					
Items	Results			Normal Ranges	Units
	2016		2017		
	Pre-operation	Post-operation			
ACTH	138.1	< 1	19.64	50–78	ng/L
PTC-8:00	887.5	51.61	304.7	147.3–609.3	Umol/L
24 h-UFC (1)	3084.5	55.6	–	110–220	IU/L
(2)	3256.0	–	–		
GH	0.23	–	–	0.126–9.88	ng/mL
PRL	25.75	34.77	ND	6.0–29.9	ng/mL
TSH	0.139	0.747	0.507	0.27–4.2	mU/l
FT3	2.82	6.01	4.19	3.6–7.5	pmol/L
FT4	13.35	14.56	15.67	12.0–22.0	pmol/L
Testosterone	0.44	–	–	Fillicular phase 2.4–12.6 Luteal phase 1.0–11.4	IU/L
DHEA-S	5.33	–	–	Fillicular phase 2.4–12.6 Luteal phase 3.5–12.5	IU/L
LH	6.3	1.6	–	Fillicular phase 1.7–7.7 Luteal phase	IU/L
FSH	6.3	2.0	–	Fillicular phase 12.4–233 Luteal phase 22.3–341	IU/L
E2		438.9	–	Fillicular phase 0.2–1.5 Luteal phase 1.7–27	IU/L
P		0.1	–		IU/L
**B. 1 mg Overnight Dexamethasone Suppression Test**					
**Items**	**Results** **(2016)**	**Results** **(2017)**	**Units**		
PTC-8:00 am	985.6	304.7	nmol/L		
PTC-(secondary 8:00 am)	959.1	19.77	nmol/L		
Suppression Rate	< 50	> 50	%		
**C. High-dose Dexamethasone Suppression Test**					
**Items**	**Results**	**Units**			
PTC-8:00 am	978.9	nmol/L			
PTC-(secondary 8:00 am)	820.3	nmol/L			
Suppression Rate	< 50	%			
**D. Inferior Petrosal Sinus Sampling and Desmopressin Stimulating Test**					
**ACTH**	**IPS**		**PV**	**IPS/PV Ratio**	
	**L (ng/L)**	**R (ng/L)**		**L**	R
0 min	233.2	1120.2	134.5	1.73	8.33
3 min	347.1	> 2000	198.7	1.75	> 10.07
5 min	307.8	1377.0	234.2	1.31	5.88
10 min	262.8	1275.0	236.7	1.11	5.39
**PRL**	**IPS**		**PV**	**IPS/PV Ratio**	
	**L (ng/L)**	**R (ng/L)**		**L**	**R**
0 min	26.35	136.3	23.44	1.12	5.83
3 min	24.25	114.3	24.48	0.99	4.67
5 min	24.62	99.53	24.92	0.99	3.99
10 min	23.95	94.64	22.9	1.05	4.13

ACTH: Adrenocorticotropic Hormone, PTC: Plasma total cortisol, IPS: Inferior Petrosal Sinus, L: left, R: right, PV: peripheral vein, PRL: prolactin, IPS/PV Ratio: The ratio of the ACTH concentration inthe inferior petrosal sinus and that in simultaneously drawn peripheral venous blood

**IPS / PV ≥ 2 (≥ 3 post CRH) –Pituitary Cushing Syndrome(Cushing’s disease, CD)**

**IPS / PV < 2 – Ectopic Cushing’s Synd**

**R-PS / L-PS ≥ 1,4 – Lateralization**

**Fig. 2:**
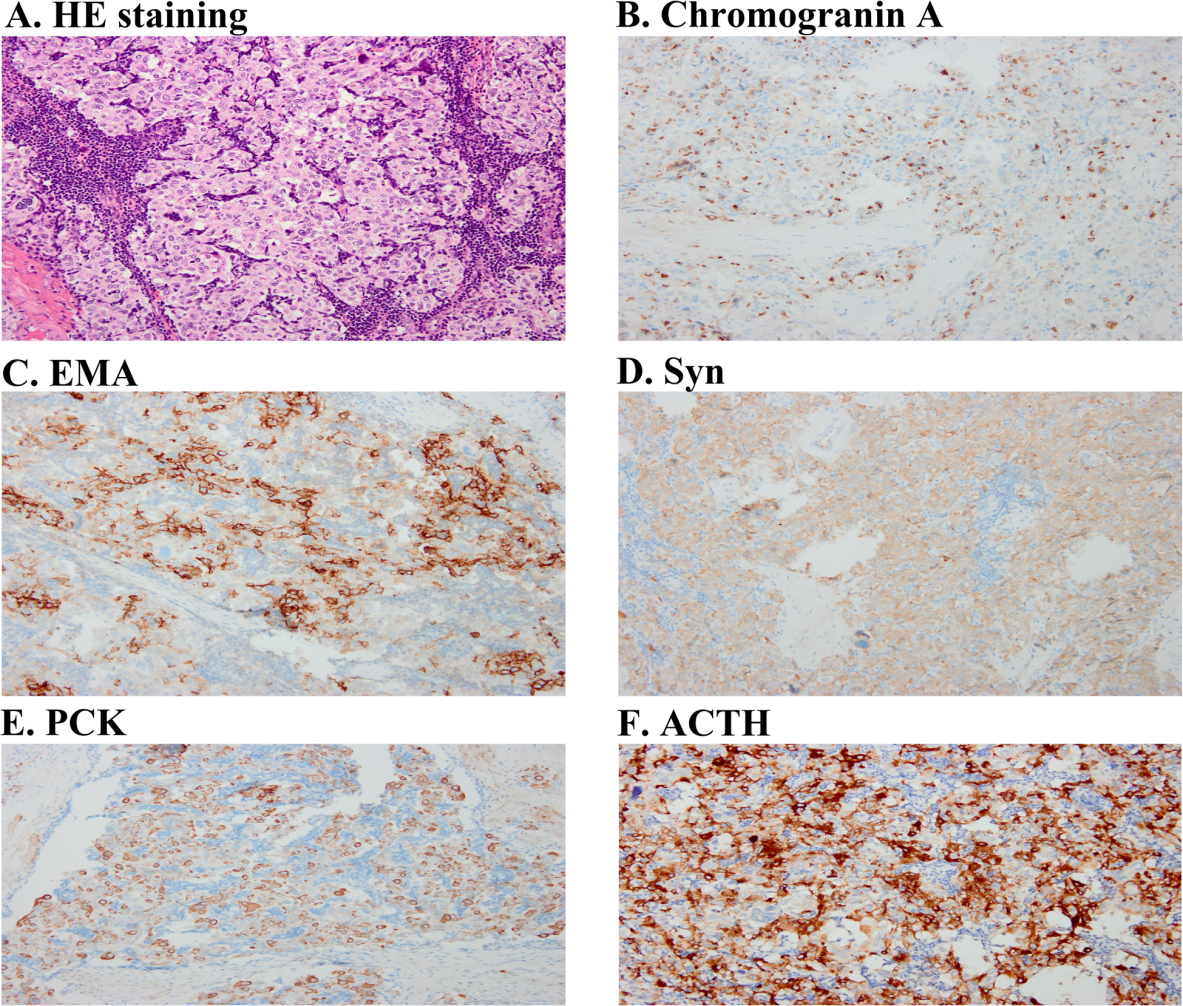
Photograph of surgical specimen. Hematoxylin and eosin (HE) stain of paraffin embedded tumor tissue (**a**) and immunochemistry with antibodies specific for Chromogranin A (**b**), EMA (**c**), Syn (**d**), PCK (**e**) and ACTH (**f**)

To find out whether orbital EAS was a possible etiology for this patient, the ophthalmologist completed the tumorectomy. The mass was sent to the pathology lab of West China Hospital. And the lesion showed positive staining of ACTH, chromogranin A, Syn, PCK and EMA (Fig. 2). According to Word Health Organism (WHO) classification 2015, the lesion was a typical orbital carcinoid tumor. Thus, the diagnosis of EAS was finally established.

The patient was discharged after surgery on replacement doses of hydrocortisone with advice on stress dosing, and with prednisone supplementation for eight weeks, followed by regular outpatient follow-up. Nine months after the operation, the patient appeared stable with no complaints nor any symptoms of CS. The ACTH and PTC levels of the patient remised to normal gradually (Table 2A, B). Post-operation MRIs were all clean (Fig. 1C). Clinical manifestations including moon face, purple striate, central obesity disappeared. The patient also reported significant improvement on life quality.

## Discussion and conclusions

The patient in our present case had an orbital mass but was asymptomatic for nearly 20 years. It took a long time and great efforts to confirm that orbital mass was actually a neuroendocrine tumor and was responsible for the case. Cushing’s syndrome is a rare disease with an estimated incidence of 0.2–5.0 out of a million people per year [1]. Endogenous Cushing’s syndrome can be divided into ACTH -depended (about 80%) and ACTH - independent (about 20%) CS [2, 3]. And the ectopic tumors are uncommon cases accounting for 10–20% of all cases of ACTH-depended Cushing’s syndrome [3, 10, 11]. Ectopic ACTH syndrome is caused by tumor cells other than pituitary gland secreting a large amount of ACTH, which stimulates adrenal gland to secrete too much cortisol and causes hypercortisolemia. According to previous reports, ectopic ACTH syndrome are mainly associated with small cell carcinoma or carcinoid of the lung, and can also be related to tumors of mediastinum, stomach, pancreases, bronchus, thymus, thyroid gland, pheochromocytoma and even reproductive system such as testicular or ovarian, but, up to 20% of them are still occult [2, 4, 5, 11–13]. More rarely, neuroendocrine tumor can produce excessive ACTH and cause EAS [14, 15]. Here, to our knowledge, we describe the first case of EAS caused by orbital neuroendocrine tumor.

EAS can affect multiple organs, therefore, localization of EAS can be challenging [16]. Nowadays, BIPSS is the gold standard test to distinguish EAS from pituitary origin with sensitivity and specificity about 95% [5, 17, 18]. Previous data have shown that an increase > 50% in ACTH and > 30% in cortisol has a very high specificity (90–100%) to potentially rule out the presence of EAS. The diagnosis of ectopic ACTH syndrome is difficult and its clinical manifestations are complex. In addition to a series of syndromes caused by Cushing’s syndrome and its complications, there are also clinical manifestations of ectopic tumor. Sometimes it is difficult to distinguish ectopic ACTH syndrome from Cushing’s disease caused by pituitary tumor, the false negative rate of MRI is 0.8%. When the differential diagnosis is difficult, it is of certain value to measure ACTH concentration by blood extraction through BIPSS. In a meta-analysis of 21 studies, the overall sensitivity and specificity of BIPSS were found to be 96 and 100% [18]. The presented case, however, showed ambiguous results, BIPSS supported a pituitary origin, while ACTH measurements, high-dose dexamethasone suppression test and MRI of sellar region indicated an extra-pituitary origin. At this point, we faced a dilemma of diagnosis. To date, there were limited reports of false positives of BIPSS of EAS [19]. It reported cyclic or relatively normal cortisol levels without suppression of corticotrophin, or ectopic CRH tumors can lead to false positive conclusions [20]. Besides, no literature presented case of orbital neuroendocrine tumor secreting ACTH [21]. In our case, pathologic findings finally helped to establish the diagnosis of EAS caused by orbital neuroendocrine tumor. According to previous study, the accuracy of lateralization is limited with a 69% positive predictive value [22]. We concluded that venous drainage between orbit and subpetrosal sinus may be the reason for the false positive results of BIPSS of our case, even though lateralization was in the opposite side. In our case, resection of the primary lesions underlying the ectopic etiology is the first-line treatment option, but when EAS is occult or metastatic, steroidogenesis inhibitors, bilateral adrenalectomy, or radiation therapy can be alternative approaches [23–26]. When the lesion of EAS is localized and not metastatic, the tumor resection can cure 76% of these patients [27].

The present study reported a rare case of orbit ectopic ACTH syndrome. The diagnosis and differential diagnosis of EAS is very challenging for clinicians. There have been extremely rare reports of ectopic ACTH syndrome in nasal or orbit origin [28]. For the patients with abnormally elevated levels of cortisol and adrenocorticotropic hormone, who was highly suspected Cushing’s syndrome, recommending function test and differential diagnosis needs to be implemented step by step according to the guidelines. DDAVP stimulation test and HDDST are important functional tests for the identification of Cushing’s disease/ectopic ACTH syndrome. When the two results are consistent, the coincidence rate for the diagnosis of Cushing’s disease or ectopic ACTH syndrome is close to 100%. Once there was paradox among the pituitary imaging reports and functional tests, BIPSS could be chose to differ Cushing’s disease and EAS. BIPSS has high sensitivity and specificity in the diagnosis of Cushing’s disease, which is the gold standard for the differential diagnosis of Cushing’s disease and EAS. Prolactin measurement and evaluation of the venogram can improve diagnostic accuracy of BIPSS [29]. But, it should be noticed that BIPSS is not perfect, anatomy structure of the sina and nearby structure especial orbital area may lead to misjudgment [30].

In summary, EAS is a rare and always challenging endocrinal disorder. The presented case is unique in that it is the first case of orbital neuroendocrine tumor originated EAS. And venous drainage between the ectopic ACTH secreting site and subpetrosal sinus can cause false positive results of BIPSS which challenge the diagnosis of EAS. And multidisciplinary team (MDT) collaboration is important for EAS diagnosis and treatment.

## Data Availability

All data generated or analyzed during this study are included within this published article. The manuscript adhered to CARE guidelines/methodology.

## Abbreviations

**ACTH**: Adrenocorticotropic hormone
**BIPSS**: Bilateral inferior petrosal sinus sampling
**CS**: Cushing’s syndrome
**CD**: Cushing’s disease
**EAS**: Ectopic adrenocorticotropic hormone syndrome
**HE**: Hematoxylin and eosin
**HDDST**: High-dose dexamethasone suppression test
**LDDST**: Low-dose dexamethasone suppression test
**MRI**: Magnetic resonance imaging
**MSCT**: Multi-slice computed tomography
**MDT**: Multi-disciplinary team
**PTC**: Plasma total cortisol
**WHO**: Word Health Organism
